# Perceptions of and barriers to family planning services in the poorest regions of Chiapas, Mexico: a qualitative study of men, women, and adolescents

**DOI:** 10.1186/s12978-017-0392-4

**Published:** 2017-10-17

**Authors:** Emily Dansereau, Alexandra Schaefer, Bernardo Hernández, Jennifer Nelson, Erin Palmisano, Diego Ríos-Zertuche, Alex Woldeab, Maria Paola Zúñiga, Emma Margarita Iriarte, Ali H. Mokdad, Charbel El Bcheraoui

**Affiliations:** 1 Institute for Health Metrics and Evaluation, 2301 5th AVE, Seattle, WA 98121 USA; 2 Salud Mesoamerica Initiative, Inter-American Development Bank, Panama City, Panama

**Keywords:** Mexico, Chiapas, Contraception, Family planning, Qualitative

## Abstract

**Background:**

In the poorest regions of Chiapas, Mexico, 50.2% of women in need of contraceptives do not use any modern method. A qualitative study was needed to design effective and culturally appropriate interventions.

**Methods:**

We used purposive maximum-variation sampling to select eight municipalities with a high proportion of residents in the poorest wealth quintile, including urban, rural, indigenous, and non-indigenous communities. We conducted 44 focus group discussions with 292 women, adolescent women, and men using semi-structured topic guides. We analyzed the data through recursive abstraction.

**Results:**

There were intergenerational and cultural gaps in the acceptability of family planning, and in some communities family planning use was greatly limited by gender roles and religious objections to contraception. Men strongly influenced family planning choices in many households, but were largely unreached by outreach and education programs due to their work hours. Respondents were aware of many modern methods but often lacked deeper knowledge and held misconceptions about long-term fertility risks posed by some hormonal methods. Acute physical side effects also dissuaded use. The implant was a new and highly acceptable method due to ease of use, low upkeep, and minimal side effects; however, it was perceived as subject to stock-outs. Adolescent women reported being refused services at health facilities and requested more reproductive health information from their parents and schools. Mass and social media are growing sources of reproductive health information.

**Conclusions:**

Our study identifies a number of barriers to family planning that have yet to be adequately addressed by existing programs in Chiapas’ poorest regions, and calls for reinvigorated efforts to provide effective, acceptable, and culturally appropriate interventions for these communities.

**Electronic supplementary material:**

The online version of this article (10.1186/s12978-017-0392-4) contains supplementary material, which is available to authorized users.

## Resumen

**Antecedentes:**

En las regiones más pobres de Chiapas, México, el 50,2% de las mujeres que necesitan anticonceptivos no utilizan ningún método moderno de planificación familiar. Ante esto, este estudio cualitativo busca generar información para diseñar intervenciones eficaces y culturalmente apropiadas.

**Metodología:**

Se utilizó un muestreo intencionado de máxima variación para seleccionar ocho municipios con una alta proporción de residentes en el quintil más pobre de la población, incluyendo comunidades urbanas, rurales, indígenas, y no indígenas. Se realizaron 44 grupos focales con 292 mujeres, adolescentes y hombres utilizando guías temáticas semi-estructuradas. Se analizaron los datos mediante abstracción recursiva.

**Resultados:**

Hubo brechas intergeneracionales y culturales en la aceptación de la planificación familiar, y en algunas comunidades el uso de la planificación familiar estuvo fuertemente limitado por los roles de género y las objeciones religiosas a la anticoncepción. Los hombres influyeron fuertemente en las decisiones de planificación familiar en muchos hogares, pero en gran medida no asistieron a sesiones educativas por conflicto con sus horarios de trabajo. Los entrevistados conocían muchos métodos modernos, pero a menudo carecían de conocimientos más profundos y tenían ideas erróneas sobre los riesgos a largo plazo en la fertilidad relacionados a algunos métodos hormonales. Los efectos secundarios agudos también desalentaron el uso de métodos hormonales. Los implantes fueron identificados como un método nuevo y altamente aceptado debido a la facilidad de uso, bajo mantenimiento, y mínimos efectos secundarios; aunque existía la percepción de que estaban sujetos a desabasto. Las adolescentes informaron haber sido rechazadas de los servicios de salud y solicitaron más información sobre salud reproductiva de sus padres y escuelas. Los medios masivos y las redes sociales son fuentes crecientes de información sobre salud reproductiva.

**Conclusiones:**

Nuestro estudio identifica una serie de obstáculos que aún no han sido abordados adecuadamente por los programas de planificación familiar existentes en las regiones más pobres de Chiapas, y subraya la necesidad de reforzar esfuerzos para proporcionar intervenciones eficaces, aceptables, y culturalmente apropiadas para estas comunidades.

## Plain English summary

In the poorest parts of Chiapas, Mexico, less than half of women who want to avoid becoming pregnant use family planning. We interviewed 292 men and women in group settings to understand why. While family planning is becoming more accepted in general, traditional gender roles and religious opposition are still challenges in some communities. Family planning is frequently framed as a women’s issue, but men strongly influence family planning choices. However, most government-sponsored educational talks are held while men are working, so family planning messages do not reach them. Most men and women know of common family planning methods but do not fully understand them. For instance, many people incorrectly worry that using methods such as birth control pills can permanently prevent them from becoming pregnant. Young and unmarried women are especially uninformed due to inadequate communication from their families and schools; they are also sometimes denied family planning services at health clinics. Many women experience strong physical side effects from using family planning methods, causing them to switch or stop entirely. Many women like the new “implant” technology, where a small device implanted in a woman’s arm prevents pregnancy for 5 years. However, health clinics sometimes do not have this or other family planning options in stock, limiting women’s abilities to make decisions about their bodies and futures. These numerous challenges have not been properly addressed by Mexico’s current family planning programs, and must be overcome to advance health and human rights for Mexico’s poorest women and communities.

## Background

While Mexico has been considered an upper-middle-income country since 1990, several states, including the state of Chiapas, still face severe poverty and poor health outcomes [1, 2]. Out of 31 Mexican states and the capital district, Chiapas has the lowest women’s life expectancy and the highest fertility rate [3, 4]. Disparities also exist within Chiapas, and the Chiapas Ministry of Health has identified 30 of Chiapas’ poorest municipalities (listed in Additional file 1: Table S1), as measured by the Human Development Index, to receive targeted interventions through the results-based Salud Mesoamerica Initiative (SMI) [5]. These municipalities are characterized by a high proportion of residents in the lowest national wealth quintile, large indigenous populations, and frequent social unrest. Most health services are provided at government facilities, which operate as a tiered network of health posts, health centers, and hospitals. Many residents participate in the Seguro Popular health insurance scheme and the Prospera conditional cash transfer program.

As part of SMI, the Chiapas Ministry of Health has committed to reducing the unmet need for contraceptives by 10 percentage points in the SMI target municipalities. This is an ambitious goal in these regions, where currently 50.2% of women in need of contraceptives are not using a modern method [6], compared to under 15% nationally [7]. Additionally, half of women have their first child before turning 19 [5], which is linked to higher maternal mortality [8], worse child health outcomes [9], and reduced educational and economic opportunities for women [10]. This is despite existing efforts to improve family planning access for vulnerable populations across Mexico, including national initiatives to reduce adolescent pregnancies [11] and the provision of free contraceptive methods through or facilitated by government clinics, Prospera, and Seguro Popular. The Salud Mesoamerica Initiative recently completed its first phase, which aimed to strengthen the supply of maternal and child health services through interventions such as supply chain strengthening and resource tracking. The second and third SMI phases target service delivery and demand generation, respectively. A qualitative study was called for in order to inform these interventions, as well as broader efforts to improve services for Chiapas’ poorest populations.

Significant quantitative work has been conducted in these impoverished communities, including a baseline household survey for SMI which provided information about women’s family planning knowledge and practices [6]. This and other quantitative studies in poor regions of Mexico have found that modern contraceptive use is significantly associated with age, number of pregnancies, receiving advice from a health provider, maternal education, household wealth, ethnicity, insurance status, female decision-making power, and delivery location [12–15]. Additionally, household surveys identified broad reported reasons for non-use, including side effects, marriage, and cultural opposition. There was also a preliminary qualitative study conducted in 2011, which assessed barriers to accessing maternal and child health services in two of Chiapas’ poorest regions, and gave several high-level findings related to family planning including women’s lack of decision-making power, fear of side effects, and experiences with method failure [16].

There is now an urgent need to understand the barriers more deeply in order to design effective and culturally appropriate interventions. For instance, what is the nature of cultural opposition, which specific barriers underlie the unmet need among adolescents, and to which side effects are women referring? This study aims to explore perceptions and identify specific barriers to the use of modern family planning methods among the poorest communities in Chiapas. The findings will help the Ministry of Health of Chiapas develop and implement tailored interventions to improve family planning services for its most vulnerable populations.

## Methods

### Methodology

This was a qualitative study based on focus group discussions (FGDs) with women and men living in municipalities with a high concentration of residents in the country’s lowest wealth quintile. Focus groups were conducted between May and October 2016.

### Development of topic guides

We developed semi-structured topic guides covering select maternal and child health topics, including family planning. The questions included open-ended prompts related to women’s and men’s knowledge, attitudes, and behaviors regarding family planning (Table 1). The same core topic guide questions relating to family planning were used in each focus group, and the interviewers were trained to probe the participants by asking additional questions to enrich the data.

**Table 1:** Focus group discussion questions related to family planning

*When is it ok for a woman to get pregnant?*
*Is it acceptable for a woman to avoid getting pregnant?*
*What could a woman do if she doesn’t want to get pregnant?*
*What are the different methods women use to prevent pregnancy?*
*If a woman decided to avoid getting pregnant, how easy would it be for her to do so?*
*How can a woman access the methods she needs to avoid pregnancy?*
*What are the obstacles that she might face when she tries to avoid pregnancy?*

### Selection of participants

We used purposive maximum-variation sampling to capture a mix of genders and ages across urban, rural, indigenous, and non-indigenous populations in Chiapas’ poorest municipalities. We first selected five rural indigenous and three urban non-indigenous municipalities with a high proportion of residents in the poorest wealth quintile: Altamirano, Salto de Agua, San Cristóbal de las Casas, San Juan Cancuc, Simojovel, Sitalá, Tecpatán, and Yajalón. These were selected from among 56 municipalities that are either receiving SMI support (30 municipalities), or serving as control districts for the evaluation of SMI, with similar socioeconomic and demographic conditions (26 municipalities).^1^ We noted no systematic differences in responses across SMI-target and control areas, and therefore present the results jointly.

We then recruited participants for four separate types of focus groups: women with children under the age of five, young women without children (who were primarily adolescents), men, and members of the community health committee. In each municipality, we held at least two FGDs of women with children, and one of each other type of FGD. We aimed to recruit eight to 12 participants per focus group, and identified eligible women and men through the health committees.

We conducted 44 focus groups with 292 total participants across the eight municipalities (Table 2). This included 19 focus groups of women with children under 5 years of age, nine focus groups of young women without children, ten focus groups of men with children, and six health committee focus groups.

**Table 2:** Characteristics of focus groups discussions and their participants

	Women with children < 5 years old	Young women without children	Men	Community health committees	Total
Total focus groups	19	9	10	6	44
Urban focus groups	8	4	4	2	18
Rural focus groups	11	5	6	4	26
Total participants	131	55	61	45	292
Median participants per group	7	6	5	7	6

There was a median of six participants in each focus group, and the size ranged from three to 13. Most respondents, especially in rural areas, had lived in their community their entire life. Rural areas were largely agricultural, while common professions in urban areas included small business owners, teachers, and physical laborers. Many respondents in rural areas came from indigenous communities that spoke Tzeltal.

### Focus group discussions

Two trained interviewers were present at each FGD. The first interviewer asked the topic guide questions and follow-up questions. After collecting the participants’ demographic information, they used the topic guide questions to lead a conversation with the participants, probing for additional relevant information based on the responses. The second interviewer was responsible for taking notes. The interviewers were all fluent in Spanish. However, several communities spoke indigenous languages and limited or no Spanish, and four focus groups were conducted primarily in an indigenous language (typically Tzeltal). In these cases, an interpreter was present to translate the interviewers’ questions and participants’ answers in real time. All FGDs were audio-recorded. On average, the interviews lasted 79 min, which included discussion of a number of maternal and child health topics, including family planning.

### Transcription and translation

The recorded interviews were transcribed verbatim and translated into English by a professional translator. Those conducted in an indigenous language were first translated to Spanish, then English. A senior bilingual faculty member on the research team systematically verified a sample of the translated transcripts for translation quality.

### Coding and analysis

Data were analyzed through a process of recursive abstraction. The responses were grouped by topic area and placed into matrices where each row was an interview question topic and each column was a focus group. One matrix was created for each of the four types of FGDs. We then assigned codes to relevant portions of the interviews as a tool to help identify themes and patterns. The codes were developed inductively from the interview data.

## Results

### Acceptability of pregnancy depends on age, economic stability, and community

Most urban focus group participants felt that society saw adolescent or unmarried pregnant women as irresponsible, and that these women and their children are sometimes rejected and harassed. Urban respondents expressed a strong interest in deferring pregnancy to allow for women’s educational or professional attainment and stability, while noting that pregnancies among older women also entailed higher risks. Participants from the rural focus groups also mentioned that unmarried mothers may be judged, but, as one rural health committee member summarized, “In this community women get married very young, and it’s normal for them to get pregnant once they’re married, perfectly normal.” Additionally, respondents in urban and rural areas held a common perception that it is irresponsible to bring a child into an impoverished family. However, several health committee and male FGD respondents stated that a government program that provides poor families with funds for every child (presumably the Prospera conditional cash transfer program, though not directly named in interviews) may incentivize some to have more children.

### Cultural opposition to family planning, despite growing acceptability

Most participants supported family planning. One commonly mentioned opinion, especially among urban women, was that use of family planning methods was a personal choice that every woman and couple should be allowed to make. Some respondents suggested that family planning was most warranted for women living in poverty or unmarried women, given that it would be irresponsible for them to become pregnant. One rural woman with children summarized, “Poor children, sometimes they don’t even have money for clothes. That is why I’m using a planning method.” Some men also expressed similar opinions: “It’s very sad when you see families with 18 children with no way to support them, children grow malnourished and they get sick.” Respondents said that individuals with substance abuse may be particularly likely not to recognize the financial responsibility of having children: “Unfortunately, we have many people who are alcoholics, and they don’t really think about that, they end up having five or more children, and since neither the man nor his wife have jobs, the poor children are left starving. They don’t stop to think about the great responsibility that children represent.”

Despite high acceptability of family planning among most participants, many knew of communities or individuals who opposed it. For instance, younger respondents saw a generational gap from older community members who rejected family planning, and urban participants said family planning was less accepted in rural, indigenous communities where women were “just meant to have children and stay at home.” While few participants expressed opposition to family planning themselves, many members of one focus group composed of rural, indigenous men stated strong cultural and religious opposition, such as, “God decided that women could get pregnant often and have as many children as they could.”

### Inconsistent findings about men as a barrier to family planning

Many women, particularly those with children, mentioned men and husbands as a main reason for women not using family planning. One urban woman with children summarized, “Many women prefer to have more children because they are afraid of their husbands.” Others stated that some men are “chauvinistic;” prevent their wives from using family planning; think family planning is a sign of cheating; or will have children with other women if their wife refuses. For instance, one rural woman with children stated: “After my first child was born, I wanted to wait before having another one, and my husband didn’t let me.” However, few men actually expressed these types of chauvinistic attitudes during the focus groups. The exceptions were several men from the rural, indigenous men’s focus group mentioned above that also expressed religious opposition.

A common theme across some respondents of both genders was that pregnancy or family planning should be a joint choice between the man and woman. It was often not explicit what this meant, but several power dynamics emerged. For instance, one rural male health committee member opined, “I think it’s OK for women to use a family planning method, but if they start making decisions on their own, that’s when problems begin.” In the same health committee FGD, a different man indicated, “In my case, I don’t want more children, and my wife has to comply.” In other instances, women said their husbands accepted their use of family planning, despite wanting more children or not wanting to space births.

### Awareness of methods does not equate to knowledge

Women with children had extensive knowledge of many temporary and permanent family planning methods, while young women without children and men were usually aware of only the most common methods, including implants, pills, injectables, and condoms. However, even if they were aware of family planning methods, women did not always have full information about them. For instance, one young woman without children explained, “I heard about the implant, I don’t know exactly what it is.”

Most women and men with children received family planning information from their doctor, often through post-partum family planning counseling. Many women with and without children had also attended community talks hosted by Prospera, the Instituto Mexicano del Seguro Social (IMSS), staff from the health center, or at their school. However, as a rural woman with children explained, the talks do not always meet women’s needs: “They came to talk to us, but we don’t know what methods are good. We are not informed.” In particular, they felt uninformed about potential side effects. Men had not typically attended talks and requested more outreach that accommodated their work schedules. Several women, including adolescents, mentioned receiving information via television or online, including through groups on social media sites.

### Supply of modern methods is usually good, but occasionally limited by stock outs and staffing

Most women with children said it was very easy for them to access family planning methods at their local health facility or pharmacy. However, a few women mentioned that their local health facility did not have implants or injectable methods in stock, and they were forced to purchase them from a private provider or pharmacy. Some participants specifically mentioned that their Seguro Popular affiliation helped them access services, but also said that lack of affiliation could be a problem for other women. Other barriers were mentioned once each: staff that do not know how to administer methods; absent specialists needed for sterilization surgery; the cost of purchasing methods from a private provider or pharmacy; and rain preventing travel to the health facility.

### Household environment limits adolescent and unmarried women’s family planning knowledge and behaviors

Many respondents felt that young and unmarried women should not be sexually active. Some young women living at home stated that having sex would be disrespectful to their parents and a pregnancy could result in being kicked out of the home. Young women without children, particularly adolescents, described family planning as a “taboo” topic they were scared to discuss with their parents. While parents were a source of information for a minority of young women without children, most felt communication on family planning was lacking, but “something families should talk about.” For instance, one urban adolescent described, “At home, my mom tells me to take care of myself but never tells me how.” Several health committees reiterated the importance of parents giving more guidance to young women, and said that many adolescents choose not to use protection despite receiving information at school. Several adolescent and young women living with their parents said it would be disrespectful to use methods such as pills while living in their parents’ homes, so they depend on male partners to use condoms.

### Stigma at health facilities and pharmacies limits adolescent girls’ access to methods

Although the supply of methods is reportedly good overall, adolescent girls face unique access barriers. Many adolescent girls feel too uncomfortable to talk to a health worker about family planning, which may be reinforced by provider behaviors. As one urban adolescent girl reported, “They only give condoms to men, not to women … At the health center, they only give underage women the talk, but no family planning methods, unless you’re married.” This concern was echoed by others who said “being underage” is a barrier to receiving methods. A rural man explained: “The problem is that when the clinic offers condoms to young people, the communities see it as giving kids permission to have sex.” Outside of health facilities, adolescent girls are nervous they may run into someone they know if attempting to purchase methods at a drugstore.

### Unwelcome side-effects and fear of long-term impacts deter women and men from hormonal methods

Many men and women had significant concerns about the short- and long-term side-effects of pills, injections, and hormonal methods overall. Short-term side effects mentioned by respondents included weight gain, mood and hormonal changes, altered menstrual cycle, headaches, nausea, and swollen feet. Many women noted that each person reacts differently, and said they dealt with problems by switching to alternate methods. However, these concerns also reduce demand, as for one rural woman with children: “It scares me, I’m afraid that [pills and injectable methods] can kill me … I prefer to have children than die of a headache because of family planning methods.”

Women and men also worried about long-term fertility impacts, including needing time to “detox” in order to become pregnant without harming the baby or miscarrying; difficulty conceiving after use; and harming or altering the uterus and ovaries. These concerns were present in both rural and urban FGDs. Several respondents cited doctors as the source of their concern. For instance, one women said the doctor recommended stopping pills after 1 year to avoid harming her body and causing infertility. Men in one rural focus group were unhappy the government is “demanding” women use family planning, as the methods can cause pain and other effects during pregnancy and delivery, and limit the effectiveness of traditional medicines administered by midwives. One man explicitly stated that he saw family planning as a “plan of the government” to weaken or kill women.

### Contraceptive preferences

Despite these concerns, when asked which methods they preferred and were most popular, most women named the implant and injectable methods, followed by the pill. The most important characteristics in a desirable family planning method were that it be long-acting with minimal upkeep, and that the woman did not have a bad physical reaction to it. Women were also concerned about pain from injections and difficulty remembering to take pills. For these reasons, a number of women preferred the implant: “The advantage of the implant is that it is inserted once and it lasts 3 years, and injections are frequent, and pills are even more frequent and if you forget to take one you can get pregnant.” However, some individuals, particularly rural men, preferred condoms or natural and herbal methods due to the side effects of hormonal methods and the perception that “chemicals are harmful.”

## Discussion

This qualitative study provides in-depth information to understand and address the family planning needs identified by prior quantitative work in the poorest regions of Chiapas, Mexico. A large and diverse group of men, women, and adolescents provided actionable insights on the influential role of culture and men in family planning; household and health system barriers affecting adolescent access to methods; and concerns about the short and long-term side effects of hormonal methods on health and fertility. This knowledge will allow the Ministry of Health and others to adapt and design targeted interventions for the most at-risk populations.

Our focus groups demonstrate the important and complex role of men in family planning. Indeed, “being married” is a barrier for nearly a quarter of poor Chiapan women with unmet family planning needs, and, accordingly, Mexico’s Specific Action Program for Family Planning and Contraception emphasizes the role of men [15, 17]. One very concerning finding is that that some women obey men’s wishes out of fear, which corroborates a prior qualitative finding that women can face physical violence for opposing their husbands [16]. This echoes sociological research describing a hegemonic Mexican society where males are a “risk factor” for women’s reproductive health [18]. However, other women in our study described a dynamic was more akin to the “relational” process described by Figueroa-Perea, whereby both sexes’ specific reproductive needs are considered and negotiated [19]. Overcoming gender barriers to family planning will require inter-sectoral action to enhance women’s rights, education, and agency to make reproductive health choices [13]. One critical action is strengthening legal and policy frameworks for gender equality, as well as accompanying monitoring and enforcement capacities [20]. Specifically, the United Nations Population Fund has called for Latin American countries to implement the Essential Services Package for Women and Girls Subject to Violence [21]. It is also critical that women actively participate in planning and implementing reproductive health programs. As successful examples from Peru and Ecuador have shown, this ensures that interventions address women’s self-identified needs and utilize local knowledge and networks [22]. While these are relatively long-term solutions, there are also short-term approaches to improving family planning access. First, health facilities can provide women with methods that can be hidden from their husbands, such as the IUD, injections, and implant. Second, family planning outreach to men can emphasize how contraceptives can help achieve their goal of supporting their families [23]. It is also important that outreach to men be scheduled to accommodate their work hours, which is not currently the case. Finally, couples-based family planning counseling has been shown effective in other developing settings, though may only be appropriate given a baseline level of gender equality [24].

A second area of need is developing targeted interventions for adolescents and young unmarried women. This is already a national priority through Mexico’s Adolescent Pregnancy Prevention Strategy, and our study offers several suggestions to strength these efforts [11]. The national approach calls for multi-sectoral action to provide youth with work opportunities and incorporate sexual and reproductive health into school curriculums. It also emphasizes ensuring access to reproductive health services; however, we find that many adolescents in poor regions of Chiapas are still embarrassed to seek care and those who do may be denied family planning methods. Further, prior studies have found that adolescents who do receive family planning care report lower service quality than women in their mid to late 20s [25]. There is a paucity of proven strategies to address these barriers. A recent review found no effects for provider training programs, and only weak effects for approaches that combined provider training, facility improvements, and broad information campaigns [26]. Therefore, innovative strategies are needed. Costa Rica is currently testing a new adolescent sexual and reproductive health strategy as part of SMI, and lessons from this experience might be applicable to Chiapas.

Another cornerstone of Mexico’s adolescent strategy is improving communication. Our findings generally support the strategy’s activities, which include improving communication with parents, providing information via text and social networks, and using popular culture and television shows to shape social norms and behaviors [11]. Within households, we found a clear need and demand for interventions to improve parent-adolescent communication about reproductive health. However, many Chiapan parents may lack critical knowledge about family planning, or be uncomfortable speaking about it with their children [27]. Therefore, proven community- and clinic-based interventions that provide parents with formal support from health care providers or health educators should be adapted and tested in the Chiapas context [28]. Out of the home, we find that post-partum family planning counseling is the most common source of information, which inherently does not reach women without children. Therefore, there is a need to implement community-based family planning activities and Chiapas can learn from regional examples such as Nicaragua’s Estrategia Comunitaria de Métodos Anticonceptivos (ECMAC) [29]. Simultaneously, our findings show that the Ministry and other programs are right to anticipate the growing role of online communication, which provides an anonymous environment for adolescents to learn about reproductive health. However, they should also be wary that these anonymous environments heighten the risk of spreading inaccurate information [30].

Many men and women expressed concerns about the side-effects of hormonal methods, corroborating quantitative evidence that side-effects are a barrier for nearly half of women with an unmet family planning need in these communities [15, 16]. These findings reinforce the need to continue developing more acceptable and lower risk family planning methods, and ensure they are accessible to the poorest women. One promising new method is the implant. While only 3.5% of Chiapan women in SMI areas had heard of this method in 2012, it is now widely known and frequently preferred according to our focus groups. It appears to be highly effective, relatively acceptable, and long lasting with minimal upkeep [31, 32]. It can also be easily hidden from men and requires less training for staff compared to the IUD [16, 33]. This is important given that one in five ambulatory facilities in SMI areas did not have staff trained to insert IUDs in a 2014 health facility survey [34]. The percent of facilities stocking implants increased from 20% in 2012 to 63% in 2014 in SMI areas, but stock outs remain a concern for women [6, 34]. The Ministry of Health must ensure that supply keeps up with demand, train sufficient providers to insert and remove the implant and consider innovative delivery mechanisms such as mobile outreach [35]. Additionally, efforts to expand availability of the implant should not detract from the provision of other methods. It is important to provide a full mix of methods so that each woman is empowered to choose the option that meets her personal reproductive needs and preferences [36]. While most focus group participants did not report being affected by stock outs, a survey of SMI health facilities found that only 67% of ambulatory-level facilities and 57% of basic-level facilities had a continuous supply of the recommended mix of methods in the 3 months prior to the survey [34]. Finally, reinvigorated efforts are needed to expand the mix of available methods by developing safer and more acceptable family planning options with fewer side effects.

In addition to the genuine risks posed by family planning methods, we also found that some perceived side effects are not supported by research and may unnecessarily dissuade contraceptive use. Specifically, cohort studies and meta-analyses do not support widespread perceptions that hormonal methods negatively affect women’s long-term fertility [37–39]. These misconceptions highlight that while many men and women are aware of family planning methods, they lack the deeper knowledge needed to make informed choices. Prospera and other programs already conduct popular family planning talks, but should revise the content to better communicate the evidence-based advantages and potential side effects of each method in language women can understand. Further, the timing of these talks should be revised to accommodate men’s work schedules. Mass media, including television, the internet, and social media, are also important channels for communicating information, particularly to adolescents. Finally, as some misconceptions were reinforced by providers, provider training on these risks is also needed. However, any interventions targeting provider-patient interactions – especially those related to conveying long-term fertility risks – must consider the difficult history in Chiapas where providers controlled poor and indigenous women’s fertility through intrauterine devices (IUDs) or sterilization without consent [40]. There is also evidence that some respondents, particularly in rural indigenous communities, continue to distrust the government and see it as a coercive force for family planning, which may reflect historical efforts to control fertility and broader political divisions in the region. Successful family planning programs will depend on employing trusted individuals to educate and provide services, and building respectful relationships with communities to overcome any underlying distrust.

This study has several limitations. First, the sampled participants may have self-selected due to an interest in health and wellness issues, meaning their knowledge and acceptance of family planning methods is higher than typical. Political affiliation may have also affected the likelihood of participating in the focus groups or biased the content of the discussions, given ongoing political unrest. However, an advantage of our study is the fact that we are an independent evaluator and not affiliated with any political group. Additionally, despite using an interpreter and translator, some portions of the transcripts from FGDs conducted in an indigenous language were disjointed and some information seems to have been lost in translation. Finally, the focus group environment may have introduced social desirability bias, and prevented some participants from expressing perspectives that would not be well-accepted by the rest of the group. Nevertheless, our study benefits from including a large number of participants, diverse in terms of age, gender, ethnicity, and location; the fact that nearly all FGD members actively participated in the conversation; and from the opportunity to triangulate our qualitative findings with recent quantitative data from the same regions.

## Conclusions

Family planning access in Chiapas’s poorest communities lags far behind the national average, and the road to overcoming this disparity is long. Family planning is becoming increasingly acceptable in Chiapan communities, though less so among older, rural, and indigenous populations. Our study identifies a number of barriers to family planning that have yet to be adequately addressed for poor Chiapan women. These include varying influences from male partners; cultural and religious barriers in indigenous communities; concerns about the side effects of contraceptive methods; misconceptions about the long-term fertility impacts of hormonal methods; inaccessibility of educational talks for men; lack of communication between parents and adolescents; and stigmatization of adolescents seeking contraception at health facilities. The findings calls for reinvigorated efforts to provide effective and culturally appropriate interventions to improve family planning access and ultimately support better health and rights for Mexico’s most vulnerable women and communities.

## Additional file

Additional file 1: Table S1. SMI Intervention and Control Municipalities. (DOCX 17 kb)

## Abbreviations

ECMAC: Estrategia Comunitaria de Métodos Anticonceptivos
FGD: Focus Group Discussion
IMSS: Instituto Mexicano del Seguro Social
IRB: Institutional Review Board
IUD: Intrauterine Devices
SMI: Salud Mesoamerica Initiative

## Footnotes

1 Salto de Agua, San Cristóbal de las Casas, San Juan Cancuc, Simojovel, Sitalá and Yajalón were selected from among 30 SMI-target municipalities, while Altamirano and Tecpatán were selected from 26 municipalities that are serving as control areas for the SMI evaluation, with similar sociodemographic profiles.
