# Supplementary material for: Perceptions of and barriers to family planning services in the poorest regions of Chiapas, Mexico: a qualitative study of men, women, and adolescents
Source: Reprod Health. 2017 Oct 17;14:129. doi: 10.1186/s12978-017-0392-4 (PMC5646150; doi:10.1186/s12978-017-0392-4)
Supplement: Additional file 1: Table S1. — SMI Intervention and Control Municipalities. (DOCX 17 kb) [file 12978_2017_392_MOESM1_ESM.docx]

Additional File 1

Table S1: SMI Intervention and Control Municipalities

| Intervention Municipalities (N=30) | Control Municipalities (N=26) |
| --- | --- |
| Aldama | Altamirano* |
| Amatenango del Valle | Benemerito de las Americas |
| Amatán | Bochil |
| Chalchihuitán | Chapultenango |
| Chamula | Coapilla |
| Chanal | Francisco León |
| Chenalhó | Ixhuatan |
| Chilón | Ixtacomitan |
| El Bosque | Ixtapa |
| Huitiupán | Ixtapangajoya |
| Huixtán | Jitotol |
| Larráinzar | Las Margaritas |
| Mitontic | Maravilla Tenejapa |
| Oxchuc | Marqués de Comillas |
| Pantelhó | Ocosingo |
| Pueblo Nuevo Solistahuacán | Ocotepec |
| Sabanilla | Ocozocoautla de Espinosa |
| Salto de Agua* | Palenque |
| San Andrés Duraznal | Pantepec |
| San Cristóbal de las Casas* | Rayon |
| San Juan Cancuc* | San Lucas |
| Santiago el Pinar | Solosuchiapa |
| Simojovel* | Soyalo |
| Sitalá* | Tapalapa |
| Tenejapa | Tecpatán* |
| Teopisca | Venustiano Carranza |
| Tila |  |
| Tumbalá |  |
| Yajalón* |  |
| Zinacantán |  |

*Included in this qualitative study
